# Outpatient Healthcare Settings and Transmission of *Clostridium difficile*

**DOI:** 10.1371/journal.pone.0070175

**Published:** 2013-07-24

**Authors:** Lucy A. Jury, Brett Sitzlar, Sirisha Kundrapu, Jennifer L. Cadnum, Kim M. Summers, Christine P. Muganda, Abhishek Deshpande, Ajay K. Sethi, Curtis J. Donskey

**Affiliations:** 1 Geriatric Research Education and Clinical Center, Cleveland Veterans Affairs Medical Center, Cleveland, Ohio, United States of America; 2 Division of Infectious Diseases, Department of Medicine, Case Western Reserve University, Cleveland, Ohio, United States of America; 3 Research Service, Cleveland Veterans Affairs Medical Center, Cleveland, Ohio, United States of America; 4 Department of Population Health Sciences, University of Wisconsin, Madison, Wisconsin, United States of America; Institute Pasteur, France

## Abstract

**Background:**

Recent reports suggest that community-associated *Clostridium difficile* infection (CDI) (i.e., no healthcare facility admission within 90 days) may be increasing in frequency. We hypothesized that outpatient clinics could be an important source for acquisition of community-associated CDI.

**Methods:**

We performed a 6-month prospective study of CDI patients to determine frequency of and risk factors for skin and environmental shedding during outpatient visits and to derive a prediction rule for positive cultures. We performed a point–prevalence culture survey to assess the frequency of *C. difficile* contamination in outpatient settings and evaluated the frequency of prior outpatient visits in patients with community-associated CDI.

**Results:**

Of 67 CDI patients studied, 54 (81%) had 1 or more outpatient visits within 12 weeks after diagnosis. Of 44 patients cultured during outpatient visits, 14 (32%) had skin contamination and 12 (27%) contaminated environmental surfaces. Decreased mobility, fecal incontinence, and treatment with non-CDI antibiotics were associated with positive cultures, whereas vancomycin taper therapy was protective. In patients not on CDI therapy, a prediction rule including incontinence or decreased mobility was 90% sensitive and 79% specific for detection of spore shedding. Of 84 clinic and emergency department rooms cultured, 12 (14%) had 1 or more contaminated environmental sites. For 33 community-associated CDI cases, 31 (94%) had an outpatient visit during the 12 weeks prior to onset of diarrhea.

**Conclusions:**

Patients with recent CDI present a significant risk for transmission of spores during outpatient visits. The outpatient setting may be an underappreciated source of community-associated CDI cases.

## Introduction

*Clostridium difficile* is the most important cause of healthcare-associated diarrhea in developed countries [1]. Although most cases of *C. difficile* infection (CDI) are acquired in hospitals or long-term care facilities [2], [3], recent reports suggest that community-associated CDI (i.e., cases with no healthcare facility admission within 90 days) may be increasing in frequency [4], [5], [6]. The source of acquisition of *C. difficile* in community-associated cases is unclear. For several reasons, it is plausible that a significant proportion of community-associated CDI cases may in actuality be healthcare-associated, but with acquisition occurring in outpatient rather than inpatient facilities. First, many patients with recently diagnosed CDI are seen in outpatient clinics after discharge from healthcare facilities. Second, patients with CDI often continue to shed spores after their diarrhea resolves [2], [7]. Current guidelines do not recommend that special precautions be taken when caring for CDI patients whose diarrhea has resolved [1]. Finally, environmental disinfection is often suboptimal in inpatient settings, and may be even less ideal in outpatient settings with frequent patient turnover.

The goal of this study was to test the hypothesis that patients with recent CDI are an underappreciated source of *C. difficile* transmission in outpatient settings. We prospectively examined the frequency of and risk factors for skin contamination and environmental shedding of spores by CDI patients at the time of outpatient clinic visits. A clinical prediction rule was derived to provide a tool to predict those patients with recent CDI presenting the greatest risk for transmission. To further assess contamination in this setting, we examined the point-prevalence of contamination of high-touch surfaces in multiple outpatient clinics and emergency departments. Finally, we determined the frequency of prior outpatient visits in all patients with community-associated CDI in our facility during a 3-year period.

## Methods

### Skin and Environmental Contamination during Outpatient Visits

The Cleveland VA Medical Center’s Institutional Review Board approved the study protocol and participants provided oral informed consent. The Institutional Review Board approved the use of an oral consent because the study was considered minimal risk. Each participant provided a signed HIPAA form which documented their participation in the study. We performed a 6-month prospective study of CDI patients seen in outpatient clinics at the Cleveland VA Medical Center. Patients were diagnosed with CDI based on presence of diarrhea and a positive commercial polymerase chain reaction (PCR) test for toxin B genes (Xpert*C. difficile*, Cepheid, Sunnyvale, CA). All patients diagnosed with initial or recurrent CDI 12 weeks or less before the clinic visit were eligible for enrollment. We chose to enroll patients up to 12 weeks after the diagnosis of CDI based on prior studies that demonstrated frequent shedding of *C. difficile* spores 1–4 weeks after completion of treatment with a subsequent reduction in shedding [7], [8]. Medical record review was performed to obtain information on demographics, medical conditions, fecal incontinence, diarrhea defined as 3 or more unformed stools within a 24 hour period, medications, and mobility subscale score from the Braden score (a score routinely calculated by the nursing staff to assess risk for development of pressure sores) [9].

Patients were seen at the time of their clinic visit in the examination room. To determine the frequency of carriage of *C. difficile*, perirectal swabs (BD BBL™ CultureSwab™, Becton Dickinson, Cockeysville, MD) were collected and cultured for toxigenic *C. difficile* as previously described [7]. To assess skin contamination, an investigator donned sterile gloves, moistened the fingertips of the gloves using sterile water, imprinted the gloves onto the patient’s abdomen, chest, arm, and hand in a standardized manner simulating a physical examination, and then imprinted the gloved hand onto a pre-reduced *C. difficile* Brucella agar (CDBA) selective plate [10]. A separate sterile glove was used to contact each culture site. To assess environmental shedding, high-touch surfaces including the examination chair arm rest, examination table, and telephone and computer keyboard in the physician work area were cultured similarly using gloved hand prints both before the provider entered the room but after collection of perirectal and skin cultures and again after completion of the outpatient visit. Patients with positive environmental cultures prior to the provider interaction were excluded from the analysis.

The CDBA plates were transferred within 15 min to an anaerobic chamber (Coy Laboratories, Grass Lake, MI) and cultures were processed as previously described [7], [10]. All isolates were tested for in vitro toxin production with use of *C. difficile*Tox A/B II (Wampole Laboratories, Inverness Medical, Princeton, NJ). Isolates that did not produce toxin were excluded from the analysis. For a subset of patients, PCR ribotyping was performed to compare perirectal, skin, and environmental isolates as previously described [11].

### Environmental Contamination in Outpatient Settings

To further assess *C. difficile* contamination in outpatient settings, we performed a point-prevalence culture survey of high-touch surfaces in 57 examination rooms in 7 outpatient clinics (3 hospital-based and 4 free-standing) and 27 examination rooms in 3 Emergency Departments (1 VA and 2 non-VA) in Northeast Ohio. The freestanding outpatient clinics provided general medical care. The hospital-based clinics provided general medical and medical and surgical subspecialty (i.e., Infectious Diseases, Geriatrics, Hematology-Oncology, Gastroenterology, Pulmonary, Rheumatology, Orthopedic Surgery, Plastic Surgery) care. Pre-moistened swabs (BD BBL™ CultureSwab™, Becton Dickinson) were applied to 3 separate sites, including the patient arm chair rest, the examination table, and the provider work area in the examination room (telephone and computer keyboard and mouse). Standardized 5×10 cm areas of the examination chair arm rest and the examination table were cultured, and the entire surface areas of the telephone, computer keyboard and mouse in the provider work area were cultured. After collection of swabs, a 2×2 cm gauze pad moistened in sterile water was applied to the same areas and placed into a sterile container. The swabs were cultured by direct plating onto pre-reduced CDBA plates and the gauze specimens were cultured by broth enrichment as previously described [7].

### Prior Outpatient Visits in Patients with Community-Associated CDI

We performed a retrospective study of all patients with community-associated CDI at the Cleveland VA Medical Center from July 2007 to June 2010. Community-associated CDI was defined as a CDI case in which symptom onset occurred in the community or 48 hours or less after admission to a healthcare facility, provided that symptom onset was more than 12 weeks after the last discharge from a healthcare facility [1]. Medical records were reviewed to determine the frequency of outpatient clinic or Emergency Department visits during the 12 weeks prior to onset of CDI symptoms.

### Data Analysis

Data were analyzed with the use of SAS statistical software, version 9.1 (SAS Institute) and STATA 11 (StataCorp, College Station, TX). To identify factors associated with shedding of *C. difficile* in the outpatient setting, bivariate analyses were performed to compare patients with positive skin and/or environmental contamination versus those with negative cultures. Student *t*- and Wilcoxon rank sum tests were used for normally and non-normally distributed data, respectively. Pearson’s chi-square and Fisher’s exact test were performed for categorical data. Using logistic regression, a prediction rule to identify CDI patients at risk for skin and/or environmental shedding was derived as previously described [12].

## Results

### Skin and Environmental Contamination during Outpatient Visits

**Figure 1:**
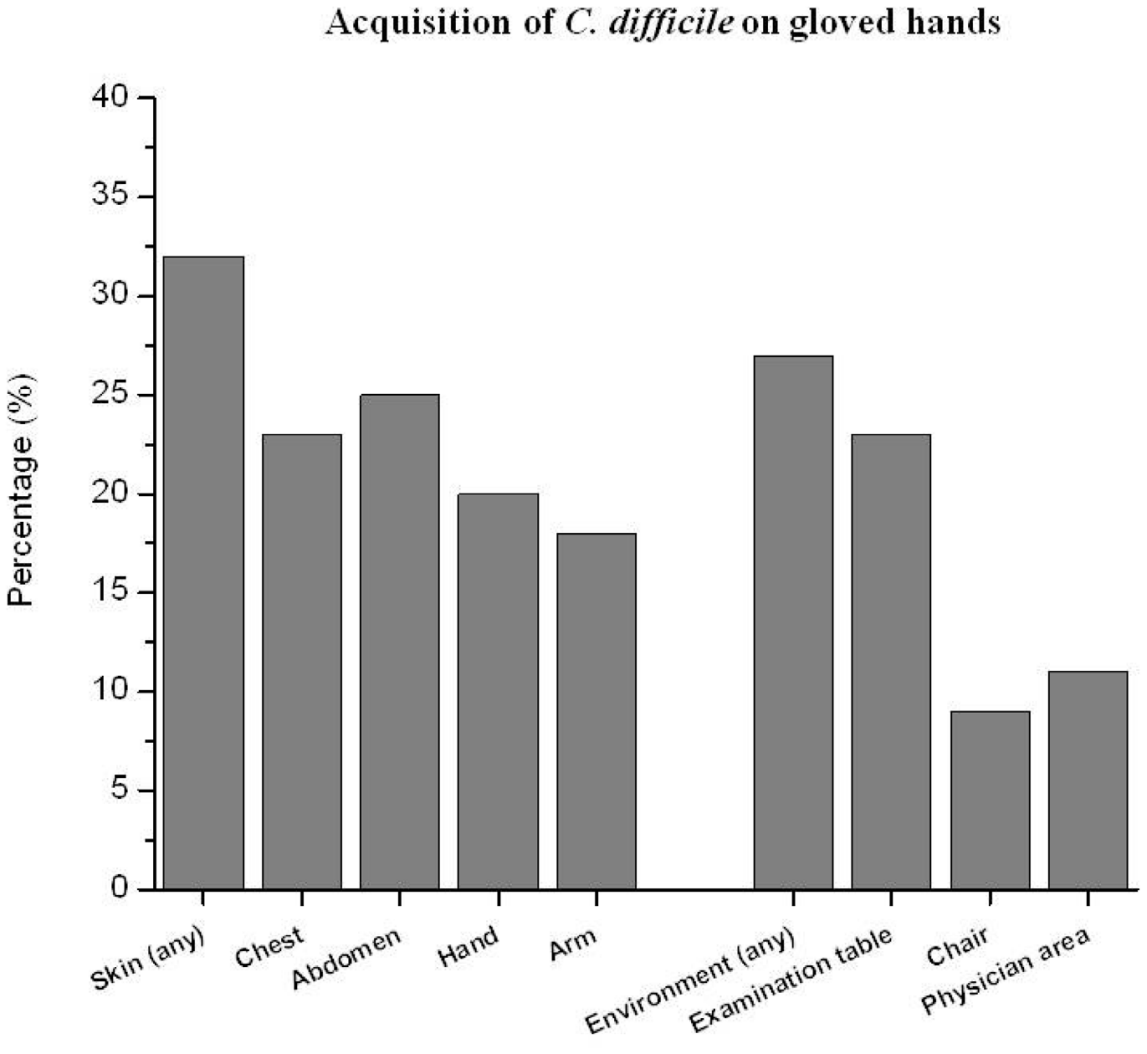
Percentage of positive gloved hand imprint cultures after contact with commonly-examined skin sites of 44 patients with recent *Clostridium difficile* infection (CDI) during outpatient clinic visits and with environmental surfaces in the examination rooms after completion of the clinic visits.

Of 67 CDI patients discharged from the hospital during the study period, 54 (81%) had 1 or more outpatient clinic visits within 12 weeks after diagnosis. Forty-four of the 54 (82%) patients with outpatient visits provided informed consent and had cultures obtained. Table 1 shows the characteristics of the patients. Of the 44 patients cultured during outpatient visits, 13 (30%) had perirectal colonization and 15 (34%) were still receiving treatment for CDI (11 receiving vancomycin tapers and 4 receiving metronidazole). Perirectal cultures were positive in 5 of 7 (7.1.4%) patients receiving non-CDI antibiotics versus 8 of 37 (21.6%) patients not receiving non-CDI antibiotics (*P* = 0.017). All of the patients on vancomycin tapers had recurrent CDI and had received 3 or more weeks of vancomycin therapy at the time of the outpatient visit. Fourteen patients (32%) had contamination of 1 or more skin sites and 12 (27%) shed spores to 1 or more high-touch surfaces in examination rooms (Figure 1); all of the patients with environmental shedding of spores also had positive skin cultures. The mean number of colonies acquired on hands after contact with the environment and skin sites were 4.8 (range, 1 to 45) and 6.5 (range, 1 to 50), respectively. Seven of the 14 (50%) patients with positive skin and/or environmental cultures had negative perirectal cultures; 4 of these 7 patients were receiving metronidazole therapy. For 5 patients, PCR ribotyping demonstrated that concurrent rectal, skin, and environmental isolates had identical banding patterns.

**Table 1:** Comparison of outpatient characteristics by skin and environmental shedding culture results.

Characteristic	All patients(N = 44)	Patients with positive skinand/or environmentalshedding cultures (N = 14)	Patients with negative skin and environmental shedding cultures (N = 30)	*P*-value
**Mean (SD) age (years)**	68.5 (11.4)	65.1 (9.9)	70 (11.9)	0.095
**Male gender**	42 (95.5)	14 (100.0)	28 (93.3)	0.460
**CDI diagnosis more than 1 month before patient visit**	31 (70.5)	9 (64.3)	22 (73.3)	0.392
**Receiving CDI therapy**	15 (34.1)	4 (28.6)	11 (36.7)	0.432
**Vancomycin taper**	11 (25)	0 (0.0)	11 (36.7)	
**Metronidazole**	4 (9.1)	4 (28.6)	0 (0.0)	
**Receiving non-CDI antimicrobials**	7 (15.9)	5 (35.7)	2 (6.7)	0.025
**Recurrent CDI**	11 (25.0)	4 (28.6)	7 (23.3)	0.490
**Incontinence**	6 (13.6)	5 (35.7)	1 (3.3)	0.009
**Diarrhea**	2 (4.5)	1 (7.1)	1 (3.3)	0.542
**Long-term care facility/Nursing home resident**	16 (36.4)	6 (42.9)	10 (33.3)	0.541
**Braden Mobility Score** *				0.000
**4**	30 (68.2)	4 (28.6)	26 (86.7)	
**3**	10 (22.7)	7 (50.0)	3 (10.0)	
**2**	4 (9.1)	3 (21.4)	1 (3.3)	
**1**	0 (0.0)	0 (0.0)	0 (0.0)	
**Perirectal swab positive for ** ***C. difficile***	13 (29.5)	7 (50.0)	6 (20.0)	0.042
**Comorbidity**				
**Diabetes Mellitus**	19 (43.2)	6 (42.9)	13 (43.3)	0.976
**Cancer**	14 (31.8)	7 (50.0)	7 (23.33)	0.077
**End-stage renal disease**	8 (18.8)	3 (21.4)	5 (16.7)	0.501
**Chronic pulmonary disease**	10 (22.7)	3 (21.4)	7 (23.3)	0.606
**Chronic neurological disease**	5 (11.4)	3 (21.4)	2 (6.7)	0.175
**Hypertension**	25 (56.8)	8 (57.1)	17 (56.7)	0.618

Notes:

Data reported as N (%) unless otherwise noted.

Percentages are based on total number of patients for each shedding group or overall as appropriate.

*P* values are based on Chi-Square or Fischer’s exact test for the categorical variables as appropriate, and on 2-sample *t* test for the continuous variable age.

* Braden mobility score (1 = Completely immobile; 2 = Very limited; 3 = Slightly limited; 4 = No limitation).

As shown in Table 1, patients with positive skin and/or environmental cultures were significantly more likely to have decreased mobility, fecal incontinence, treatment with non-CDI antibiotics, and current metronidazole CDI treatment than patients with negative skin and/or environmental cultures. Current treatment with a vancomycin taper was protective (i.e., none of the 11 patients receiving vancomycin taper had positive skin or environmental cultures). None of the patients on current CDI therapy had diarrhea. Two patients not on CDI therapy had diarrhea, including one patient with positive skin and environmental cultures who was diagnosed with recurrent CDI at the time of the visit and one patient with negative skin and environmental cultures whose diarrhea was attributed to cancer chemotherapy. For patients not on CDI therapy, a prediction rule including having either incontinence or decreased mobility was 90% sensitive and 79% specific for detection of shedding of spores (Table 2).

**Table 2:** Prediction rules for skin/environmental shedding among 44 patients receiving versus not receiving CDI therapy at the time of the cultures of whom 14 (31.8%) had positive skin and/or environmental cultures for *C. difficile.*

Prediction rule	Sensitivity to identify patients with positive skin and/or environmental shedding cultures	Specificity to identify patients with negative skin and environmental shedding cultures	Proportion of all patients correctly predicted as shedders or non-shedders	OR (95% CI)
**Patients receiving current CDI therapy (N = 15)**	N = 4	N = 11	N = 15	
**CDI therapy with metronidazole (vs. vancomycin taper)**	4/4 on metronidazole (100.0%)	11/11 on vancomycin taper (100.0%)	15/15 (100.0%)	Perfect prediction
**Patients not receiving current CDI therapy (N = 29)**	N = 10	N = 19	N = 29	
**Incontinence present (vs. absent)**	4/10(40.0%)	18/19(94.7%)	22/29(75.9%)	14.6(1.1, 129.4)
**Incontinence OR decreased mobility (Braden score <4)** * **present (vs. both absent)**	9/10(90.0%)	15/19(79.0%)	24/29(82.8%)	33.8(3.2, 351.0)

* Braden mobility score (1 = Completely immobile; 2 = Very limited; 3 = Slightly limited; 4 = No limitation).

### Environmental Contamination in Outpatient Settings

Toxigenic *C. difficile* was detected in 2 of 3 Emergency Departments, 3 of 4 freestanding outpatient clinics, and 3 of 3 hospital-based clinics (Table 3). Of 84 total rooms cultured, 12 (14%) had 1 or more contaminated environmental sites. Nine of 165 (5%) outpatient clinic sites in 9 different rooms were contaminated, with the provider’s work area and the patient’s examination table being most frequently contaminated. Three of 81 (4%) Emergency Department sites in 3 different rooms were contaminated, with the patient's bed being most frequently contaminated. All of the positive cultures were obtained by broth enrichment, whereas no samples collected using swabs that were directly plated were positive.

**Table 3:** Point-Prevalence of Environmental Contamination with *Clostridium difficile* in Examination Rooms in Outpatient Clinics, Emergency Departments and Specialty Clinics.

	Rooms N (%)	Sites N (%)
**Total**	12/84 (14)	12/252 (5)
**Outpatient Clinic**	8/42 (19)	8/126 (6)
**Emergency Department**	3/27 (11)	3/81(4)
**Specialty Clinics**	1/15 (7)	1/45 (2)

### Prior Outpatient Visits in Patients with Community-Associated CDI

During the 3-year period studied, 33 of 244 (14%) total CDI cases were classified as community-associated cases. Table 4 shows the characteristics of the 33 patients with community-associated CDI. Thirty-one of 33 (94%) had 1 or more outpatient healthcare visits during the 12 weeks prior to onset of diarrhea. The median number of outpatient visits was 5 (range, 1 to 21).

**Table 4:** Characteristics of 33 Patients with Community-Associated *Clostridium difficile* Infection.

Characteristic	Subjects (N = 33)
**Mean age, y (range)**	67 (27–90)
**Male sex**	31 (94%)
**Female sex**	2 (6%)
**Medical Conditions**	
**Diabetes mellitus**	13 (39%)
**Cancer**	3 (9%)
**End-stage renal disease**	3 (9%)
**Chronic pulmonary disease**	4 (12%)
**Chronic neurologic disease**	3 (9%)
**Outpatient visits in past 12 weeks**	31 (94%)
**Antibiotics in the past 12 weeks**	22 (67%)
**Mean outpatient visits in the past** **12 weeks (range)**	5 (1–21)

Data are no. (%) of patients, unless otherwise specified.

## Discussion

Our study provides support for the hypothesis that the outpatient healthcare setting may be an underappreciated source of community-associated CDI cases. First, we found that 81% of CDI patients discharged from the hospital had 1 or more outpatient clinic visits within 12 weeks after discharge. About one-third of these patients presented a risk for transmission based on acquisition of spores on investigators’ hands after contacting frequently examined skin sites and/or high-touch environmental surfaces after completion of the outpatient visit. These data are consistent with a recent study demonstrating that about half of inpatients with CDI continue to shed spores for up to 4 weeks after completion of CDI therapy [7]. Second, a point-prevalence culture survey of outpatient clinics and Emergency Departments in Northeast Ohio demonstrated that 14% of rooms had positive cultures for toxigenic *C. difficile*. Finally, we found that 94% of cases of community-associated CDI from our institution had outpatient healthcare facility visits during the 12 weeks prior to onset of diarrhea (range, 1 to 21 visits).

None of the 11 patients receiving treatment with a vancomycin taper had positive rectal, skin, or environmental cultures, suggesting that the prolonged tapers of vancomycin maintain suppression of *C. difficile* in the intestinal tract. All of these patients had received at least 3 weeks of vancomycin therapy at the time of their outpatient visit. In contrast, all 4 of the patients receiving treatment with metronidazole had positive skin and/or environmental cultures. All of these patients had received <10 days of metronidazole at the time of the outpatient visit, consistent with recent evidence that many patients continue to shed spores during initial courses of CDI therapy with vancomycin or metronidazole [7]. For patients not on CDI therapy, decreased mobility, fecal incontinence, and treatment with non-CDI antibiotics were associated with positive skin and/or environmental cultures. Diarrhea and fecal incontinence have been associated with shedding of other pathogens [13], and it is plausible that they contribute to shedding of *C. difficile* spores [14]. Use of non-CDI antibiotics is a major risk factor for recurrence of *C. difficile* [15] and may also contribute to shedding of spores in the absence of overt CDI by promoting overgrowth of *C. difficile* in the intestinal tract. The reason for the association between decreased mobility and skin contamination and environmental shedding is not clear, but one possible explanation could be that individuals with decreased mobility have less ability to bathe effectively. For patients not on CDI therapy, a prediction rule including incontinence or decreased mobility was 90% sensitive and 79% specific for detection of shedding of spores (Table 2).

**Figure 2:**
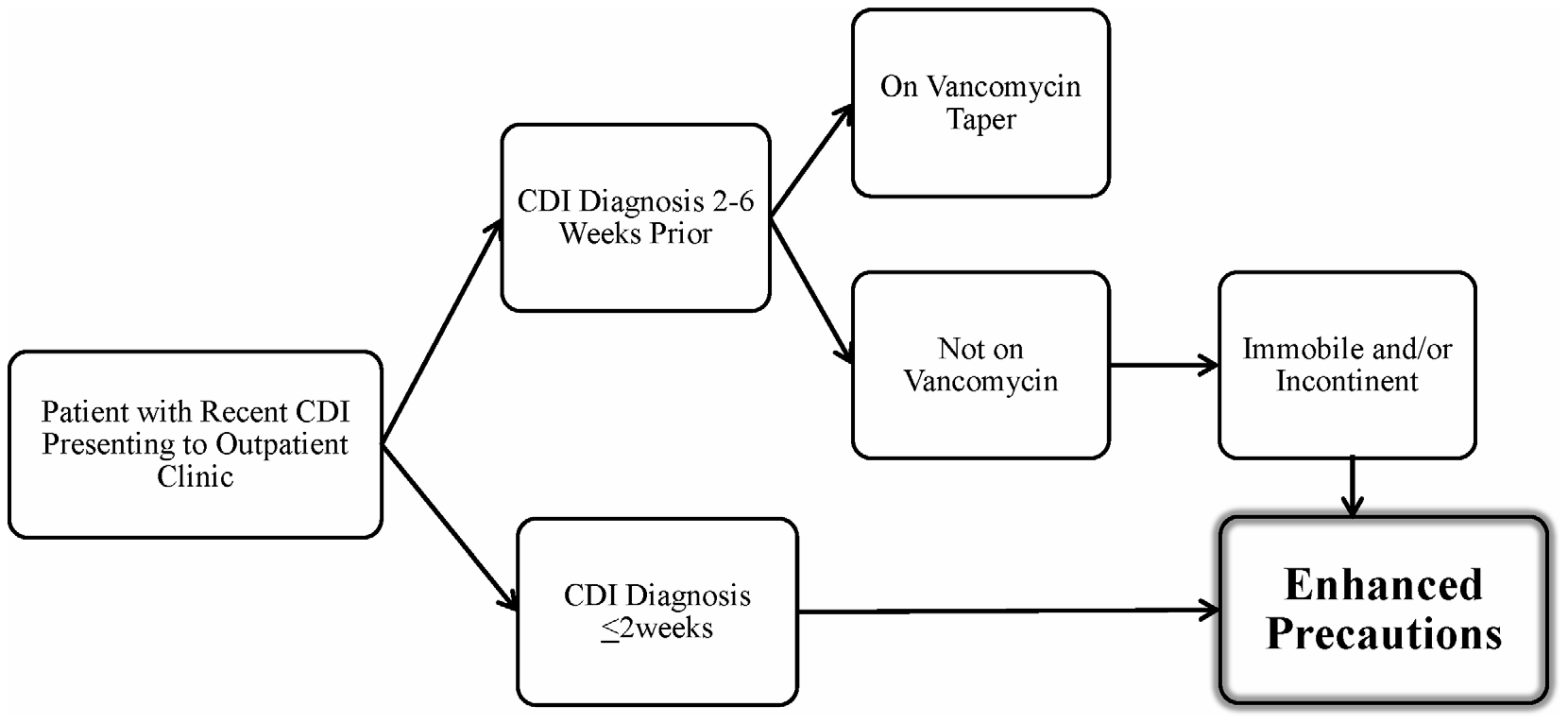
Proposed algorithm for management of patients with recent *Clostridium difficile* infection (CDI) presenting to outpatient clinics. Enhanced precautions includes wearing gloves when examining patients and cleaning high-touch surfaces with sporicidal disinfectants after visits.

Our findings have important implications for infection control of C. *difficile* in outpatient settings. Clinicians should be aware that patients with recent CDI may have skin contamination and that spores may be spread to environmental surfaces during outpatient visits. Based on our findings, figure 2 provides a proposed algorithm for management of patients with recent CDI presenting to outpatient clinics. Patients on CDI therapy for ≤2 weeks are at high risk for transmission, particularly during the first few days of therapy [7], and should be managed with enhanced precautions including wearing gloves when examining patients and cleaning high-touch surfaces with sporicidal disinfectants after visits. Similarly, patients diagnosed with CDI in the past 2–12 weeks but not on current therapy should be managed with enhanced precautions if they are immobile or have fecal incontinence. Such measures might be particularly indicated in clinics where many patients are at risk for CDI due to antibiotic therapy (e.g., oncology and infectious diseases clinics). Given the small sample size of our study, the proposed algorithm should be considered preliminary and further studies will be needed for validation.

The finding that patients with community-associated CDI had frequent exposures to outpatient healthcare facilities is consistent with 2 other recent publications. Kutty et al. [5] found that more than half of patients with community-associated CDI in both VA and non-VA facilities in North Carolina had >1 outpatient visit, and recent outpatient visits were significantly associated with community-associated CDI in the VA population. Similarly, Dumyati et al. [6] reported that 69% of patients with community-associated CDI had received care at an outpatient physician office in the 12 weeks before the diagnosis of CDI, and 83% had received outpatient care when dental offices and Emergency Department visits were included.

We found that 67% of patients with community-associated CDI had received antibiotics in the prior 12 weeks. This percentage is similar to previous reports that 40% to 76% of patients with community-associated CDI have prior documentation of antibiotic exposure [5], [6], [16], [17]. These data suggest that antimicrobial exposure plays a major role in community-associated CDI and efforts are needed to limit inappropriate antimicrobial prescription in this setting. However, it is notable that nearly one-third of patients with community-associated CDI had no documented prior antimicrobial exposure. Other factors have been associated with community-associated CDI, including gastric acid suppressants, exposure to infants, and exposure to family members with CDI [5], [6], [18]. These factors were not evaluated in the current study.

Our study has several limitations. First, the study population for the evaluation of skin and environmental contamination included mostly elderly men. Additional studies are needed in other patient populations. Second, the small sample size limited the assessment of risk factors for shedding. However, the factors associated with shedding (i.e., decreased mobility and fecal incontinence) or absence of shedding (i.e., vancomycin taper therapy) are plausible and consistent with previous studies [7], [11]–[12]. Although only 4 patients receiving current metronidazole for CDI therapy were assessed, our findings are consistent with previous studies that have demonstrated that shedding of spores is common during metronidazole therapy [7], [19]. As noted previously, the prediction rule is limited due to the small sample size and larger studies are needed to validate the rule. Finally, the fact that many outpatient clinics had *C. difficile* contamination and most patients with community-CDI had 1 or more outpatient visits provides suggestive, but not definitive, evidence that the outpatient facilities may have been the site of acquisition. Additional studies that include molecular typing of isolates from outpatient clinics and from patients who develop community-associated CDI after visiting those clinics will be necessary to prove a definite link between outpatient clinics and CDI cases.
